# Host Immunogenetics and Chronic HCV Infection Shape Atopic Risk in Pediatric Beta-Thalassemia: A Genotype–Phenotype Study

**DOI:** 10.3390/genes16121440

**Published:** 2025-12-02

**Authors:** Caterina Cuppari, Alessio Mancuso, Laura Colavita, Clelia Cusmano, Valeria Tallarico, Valerio Caruso, Roberto Chimenz, Mimma Caloiero, Mariarosa Calafiore, Antonina La Mazza, Luciana Rigoli

**Affiliations:** 1 Pediatric Emergency Unit, Department of Maternal and Child Health, University Hospital of Messina, “G. Martino” Policlinic, 98124 Messina, Italy; 2 Department of Human Pathology in Adult and Developmental Age “Gaetano Barresi”, University of Messina, “G. Martino” Policlinic, 98124 Messina, Italy; 3 Department of Pediatric, Pugliese-Ciaccio Hospital, 88100 Catanzaro, Italy; valeriatallarico.ped@gmail.com; 4 Psychiatry 2 Unit, Clinical and Experimental Medicine Department, University of Pisa, 56126 Pisa, Italy; 5 Pediatric Nephrology and Dialysis Unit, University Hospital “G. Martino”, 98124 Messina, Italy; 6 Department of Pediatrics, Lamezia Terme Hospital, 88046 Lamezia Terme, Italy; 7 Department of Pediatrics, Polistena Hospital, 89024 Polistena, Italy; 8 Department of Human Pathology of Adulthood and Childhood “G. Barresi”, University of Messina, 98125 Messina, Italy

**Keywords:** HCV, IL10, TLR7, atopy, thalassemia, pediatric immunity, IgE, immune regulation, chronic infection, gene–environment interaction

## Abstract

Background: Pediatric patients with beta-thalassemia (BT) face unique immunologic challenges due to chronic transfusions and viral exposure. Hepatitis C virus (HCV), a common infection in polytransfused individuals, may influence immune polarization. However, the combined effect of chronic HCV and host immunogenetics on allergic sensitization remains incompletely understood. Objective: To assess total serum IgE levels and allergic manifestations in HCV-positive vs. HCV-negative BT patients, and explore associations with common polymorphisms in IL10, TLR7, IL4, and IFNG genes Methods: This cross-sectional observational study enrolled 46 BT patients (37 HCV-positive, 9 HCV-negative) and 50 healthy controls. Clinical allergy history, total IgE levels (ELISA), and skin prick tests (SPT) for aeroallergens were collected. Genotyping for IL10 −1082, TLR7 rs179008, IL4 −589, and IFNG +874 polymorphisms was performed. Associations between genotypes, HCV status, and IgE levels were analyzed descriptively due to small sample size Results: HCV-positive BT patients had lower mean IgE levels (18.73 ± 4.2 IU/mL) and fewer reported allergic symptoms (21.6%) compared to HCV-negative counterparts (118.76 ± 7.9 IU/mL; 55.5%). The IL10 −1082 AA and TLR7 rs179008 TT genotypes were more common in the HCV-positive group and were associated with lower IgE levels. No associations were noted for IL4 or IFNG variants. Splenectomy appeared to further modify IgE levels in HCV-negative patients. Due to limited power and absence of multivariate analysis, findings are exploratory. These preliminary observations may inform future studies of immune deviation in chronically infected pediatric cohorts. Conclusions: Chronic HCV infection may contribute to immune tolerance and reduced allergic expression in BT patients, potentially modulated by IL10 and TLR7 genotypes. Further studies with functional immune profiling and larger cohorts are required.

## 1. Introduction

Beta-thalassemia major is a hereditary hemoglobinopathy characterized by ineffective erythropoiesis and severe anemia, often requiring lifelong red blood cell transfusions [1]. These repeated transfusions, while lifesaving, predispose patients to a range of complications, including iron overload, transfusion-transmitted infections, and altered immune function [2]. Among the most clinically relevant of these complications is hepatitis C virus (HCV) infection, which remains prevalent in multi-transfused populations despite improvements in blood screening procedures [3]. HCV infection not only contributes to hepatic and systemic morbidity but also has profound effects on host immunity [4]. Chronic HCV infection is known to modulate immune responses through the induction of regulatory pathways, including those mediated by interleukin-10 (IL-10), which play a key role in promoting immune tolerance [5]. In parallel, patients with beta-thalassemia exhibit a distinct immunological profile due to both their underlying genetic disorder and the immunomodulatory consequences of chronic transfusion therapy [6,7]. Dysregulation of T cell subsets, impaired B cell maturation, and altered cytokine secretion are common findings in this patient population [3].

Additionally, splenectomy, which is often performed to manage hypersplenism or to reduce transfusion requirements, further complicates immune surveillance by removing a key site for antigen processing and immune regulation. Collectively, these factors create a unique immunological environment that may influence the development of hypersensitivity disorders such as atopy [8,9,10,11]. Atopic diseases, defined by the presence of elevated serum IgE levels, allergen sensitization, and clinical symptoms such as asthma, rhinitis, or eczema, are the result of complex interactions between genetic predisposition and environmental exposures. Genetic polymorphisms in immune regulatory genes, such as IL10 and Toll-like receptor 7 (TLR7), have been implicated in modulating the Th1/Th2 balance and influencing susceptibility to allergic diseases. IL-10 is a critical anti-inflammatory cytokine that inhibits both Th1 and Th2 responses under specific conditions and contributes to peripheral tolerance by promoting regulatory T cell activity. TLR7, an endosomal receptor involved in the recognition of single-stranded RNA viruses, plays an essential role in antiviral immunity and may influence cytokine production and immune polarization following chronic viral stimulation [12].

Emerging evidence suggests that persistent infections, including helminths and chronic viral pathogens, may reduce the risk of atopy through the induction of IL-10 mediated immune deviation or suppression. In this context, chronic HCV infection presents a biologically plausible model for studying infection-induced immune tolerance and its implications for allergic disease. While viral infections are generally associated with enhanced inflammation and immune activation, the persistent nature of HCV infection may promote a unique immunological adaptation. This adaptation could lead to suppression of hypersensitivity responses, either through the expansion of regulatory T cells or through shifts in the cytokine milieu toward a more tolerogenic profile [13]. This immune deviation has also been observed in other pediatric conditions [14,15,16,17]. The immunogenetic context of the host plays a critical role in modulating these interactions. The IL10 gene is highly polymorphic, and different promoter variants are known to affect IL-10 expression levels. The −1082 AA genotype has been associated with low IL-10 production in several populations, though its effect can vary depending on the immune challenge or pathological condition. Similarly, TLR7, encoded on the X chromosome, exhibits functional polymorphisms that may influence antiviral signaling. The rs179008 polymorphism, in particular, has been studied for its impact on TLR7 translation and its differential effect in males and females. These genetic factors may determine how an individual responds to chronic HCV infection and whether this response tilts the balance toward immune suppression or activation [18,19,20,21]. Despite the theoretical underpinnings of this relationship, few studies have explored the clinical implications of chronic HCV infection on allergic outcomes in patients with genetic predispositions to altered immune responses. Pediatric beta-thalassemia patients represent an ideal population in which to study these phenomena, as they are frequently exposed to HCV through transfusions and often undergo splenectomy, creating a multifactorial model of immune dysregulation [22]. The intersection of chronic viral infection, genetic background, and iatrogenic immune modulation provides a rich framework for investigating novel mechanisms of allergy regulation.

The objective of this study was to investigate the association between HCV infection, genetic variants in IL10 and TLR7, and the occurrence of atopy in a cohort of polytransfused pediatric beta-thalassemia patients. We hypothesized that chronic HCV infection may confer protection against allergic manifestations by promoting a tolerogenic immune profile, and that this effect may be influenced by host genetic factors. By examining total serum IgE levels, skin prick test results, transfusion history, splenectomy status, and genotypic distribution, we sought to characterize the immunogenetic landscape underlying atopic risk in this unique population. Our findings aim to contribute to a deeper understanding of gene–environment interactions in chronic disease settings and may offer new insights into the immunological consequences of persistent viral infections.

## 2. Materials and Methods

### 2.1. Study Design and Participants

We conducted a cross-sectional observational study involving pediatric and young adult patients diagnosed with beta-thalassemia major, managed at a tertiary referral center specializing in hemoglobinopathies. The inclusion criteria were: age between 10 and 25 years, confirmed diagnosis of transfusion-dependent BT, and regular red blood cell transfusions for a minimum of 5 years. Patients were excluded if they had received immunosuppressive therapy, undergone hematopoietic stem cell transplantation, or had a known diagnosis of primary immunodeficiency. Classification of atopy was based on a combination of clinical allergic history (including symptoms such as urticaria, rhinitis, or asthma), total serum IgE levels, and positive skin prick test reactivity to at least one common aeroallergen. The study cohort comprised 46 BT patients, of whom 37 tested positive for anti-HCV antibodies and 9 were seronegative. A control group of 50 healthy individuals with no history of chronic illness or transfusion was included for baseline comparisons. Informed consent was obtained from all participants or their legal guardians. The study was approved by the local ethics committee and conducted in accordance with the Declaration of Helsinki.

### 2.2. Clinical and Allergy Assessment

Detailed medical histories were obtained, including age at diagnosis, transfusion schedule, splenectomy status, family history of atopy, personal history of allergic rhinitis, asthma, eczema, or post-transfusion urticarial reactions. Nutritional status, serum ferritin, and liver enzyme levels were retrieved from clinical records. Skin prick testing (SPT) was performed using standardized extracts (including dust mite, grass pollen, tree pollen, cat, dog, and Alternaria). Wheals ≥ 5 mm after 15 min were considered positive. All SPTs were conducted by trained personnel under the supervision of an allergist.

### 2.3. IgE Quantification

Total serum IgE was measured using a commercial ELISA kit (AccuBind ELISA Microwells; Monobind, Lake Forest, CA, USA), with a normal reference range defined as 0–200 IU/mL. Samples were processed in duplicate and assays were repeated if the coefficient of variation exceeded 10%. Measurements were obtained at least 14 days post-transfusion to reduce confounding by transient immune activation.

### 2.4. Virological Testing

Serologic testing for HCV antibodies was performed using the Abbott HCV EIA 2.0 platform. Quantification of HCV RNA was performed with Bayer Quantiplex HCV RNA 2.0 assay. In patients with detectable viral load, genotype determination was conducted using restriction fragment length polymorphism (RFLP) methods. Where available, liver fibrosis staging was reviewed via FibroScan or biopsy reports.

### 2.5. Genotyping Procedures

Genomic DNA was extracted from peripheral blood leukocytes using phenol-chloroform extraction. Genotyping for IL10 −1082 (rs1800896), TLR7 rs179008, IL4 −589 (rs2243250), and interferon-gamma (IFNG) +874 (rs2430561) was performed using PCR amplification followed by digestion with specific restriction enzymes. Electrophoretic separation of digested fragments was used for allele discrimination. A subset of samples underwent confirmatory Sanger sequencing. Genotype frequencies were compared to public databases for European ancestry controls.

### 2.6. Statistical Analysis

Given the limited number of participants, analyses were primarily descriptive. Categorical variables were compared using Fisher’s exact test, and continuous variables with Student’s *t*-test. Genotype distributions were tested for Hardy–Weinberg equilibrium. No multivariate models or adjustments for multiple comparisons were performed due to sample size constraints. Results were interpreted cautiously, with emphasis on observed trends rather than statistical significance.

## 3. Results

Among the 46 beta-thalassemic patients included in the study, 37 were HCV-positive (mean age 32), while 9 were HCV-negative (mean age 20). Of the HCV-positive group, 19 were HCV RNA-positive, indicating active infection, while the remaining 18 were HCV RNA-negative. Splenectomy had been performed in 26 patients, whereas 20 patients retained their spleens. The distribution of splenectomy was similar across HCV-positive and HCV-negative subgroups, allowing for assessment of splenic status as an independent immunomodulatory factor.

Mean serum IgE levels were significantly lower in HCV-positive than in HCV-negative thalassaemic patients (18.73 ± 4.2 vs. 118.76 ± 7.9 IU/mL; *p* < 0.001, Student’s *t*-test). Both patient subgroups differed significantly from healthy controls (*p* < 0.05, ANOVA), suggesting a robust inverse association between HCV infection and Th2-driven atopic responses. Within the HCV-positive group, no substantial difference in IgE levels was observed between HCV RNA-positive (mean: 17.2 IU/mL) and HCV RNA-negative (mean: 17.26 IU/mL) subgroups, suggesting that the immunomodulatory influence of HCV may persist even in the absence of active viral replication. This finding implies that chronic antigenic exposure, rather than viremia per se, may be the critical factor modulating immune tolerance. Interestingly, previous studies have shown that chronic infections can shape immune deviation, particularly when modulated by IL-10 and TLR signaling pathways. For instance, gene–environment interactions have been implicated in inflammatory and immunological responses in numerous other pediatric conditions [23,24,25,26,27,28,29].

Allergic history and transfusion-related urticarial reactions were also evaluated. Among HCV-negative patients, 5 out of 9 (55.5%) had a history of urticaria, and 3 of these patients showed the highest serum IgE levels. In contrast, only 8 out of 37 (21.6%) In Results (page 9, same paragraph as IgE results), after your IgE comparison, add:

The proportion of patients with allergic manifestations was significantly lower in HCV-positive than in HCV-negative β-thalassaemia patients (21.6% vs. 55.5%; *p* < 0.05, Fisher’s exact test). HCV-positive patients reported urticarial symptoms, and just 3 experienced transfusion reactions involving systemic symptoms such as tremors or fever. Among HCV-positive patients, only 5 (13.5%) had a history of allergic rhinitis with positive skin test results, compared to 4 out of 9 (44.4%) in the HCV-negative group. This highlights the reduced prevalence of both subjective and objective indicators of allergy among patients chronically exposed to HCV.

Skin prick testing confirmed sensitization in patients with elevated IgE and respiratory symptoms. The four individuals with the highest IgE levels and confirmed skin test positivity had clinical presentations consistent with atopic disease (two had asthma, one rhinitis, and one conjunctivitis/rhinitis). All four of these patients were HCV-negative, splenectomized, and had no family history of atopy, suggesting that environmental and disease-related immune modulation may be significant contributors. This subgroup represents a phenotype of “non-heritable atopy,” potentially driven by the combined immunological effects of splenectomy and absence of chronic viral modulation. Similar environmental triggers have been reported to induce atypical immune profiles in pediatric cardiopathies and mitochondrial dysfunction [30] reinforcing the idea that secondary immune changes can mimic primary atopic predisposition. Splenectomy status further influenced IgE levels. Splenectomized, HCV-negative patients showed the highest serum IgE levels among all subgroups. The mean IgE value in patients who experienced urticarial reactions after transfusion was 81 IU/mL, versus 21 IU/mL in those without such reactions, indicating a potential link between immune dysregulation post-splenectomy and allergic predisposition. In contrast, splenectomized HCV-positive patients had lower mean IgE values than their HCV-negative counterparts, highlighting a potential mitigating effect of chronic HCV exposure on Th2-mediated responses. These patterns were consistent even after stratifying for clinical history of allergic symptoms and SPT results.

The control group of 50 healthy individuals had a mean serum IgE value of 16.84 IU/mL, falling within the expected normal range and corroborating the elevated values observed in the HCV-negative beta-thalassemia subgroup. While allergic symptoms were not systematically recorded in the control group, the observed IgE levels support the use of this group as a baseline for interpreting immune deviations in the thalassemic population.

Genotypic analysis further contextualized the immunological findings. A higher prevalence of IL10 −1082 AA and TLR7 rs179008 TT genotypes was observed among HCV-positive patients with low IgE levels Table 1, Table 2 and Table 3. Although not powered for statistical significance, this trend suggests a possible gene-virus interaction in shaping the immune profile. The presence of these genotypes may potentiate or sustain IL-10 production under chronic viral exposure, contributing to immune tolerance and Th2 suppression. This hypothesis aligns with emerging models of viral-immunogenetic synergy observed in other pediatric inflammatory conditions and congenital immunopathologies [24,27,31,32,33]. Further studies with larger cohorts are necessary to validate these observations and evaluate gene–environment synergy through multivariate modeling.

**Figure 1:**
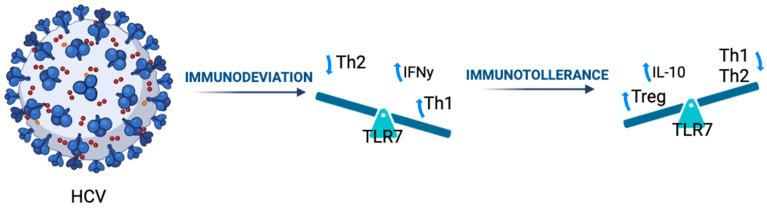
Possible role of HCV infection in thalassemic patients. Schematic representation of immune modulation associated with chronic HCV infection in β-thalassaemic patients. The diagram illustrates the progression from Th2-dominant immunodeviation (↓ Th2, ↑ Th1, ↑ IFNγ) toward regulatory immunotolerance (↑ Treg, ↑ IL-10, ↓ Th1, ↓ Th2) mediated through TLR7 activation.

Collectively, these results support a model wherein chronic HCV infection, especially in the presence of specific immunogenetic backgrounds, may induce a regulatory immune state that counteracts the development of allergic sensitization and IgE-mediated responses in beta-thalassemic individuals. See Figure 1.

**Table 3:** Correlation of Serum IgE with Clinical Parameters in BT Patients. Comparisons performed using independent-sample *t*-tests or Mann–Whitney U tests, as appropriate. *p* < 0.05 considered statistically significant; ns = not significant. Data for splenectomised HCV-negative patients are descriptive only due to very small sample size.

Parameter	Subgroup	Mean IgE (IU/mL)	Comparison
HCV RNA	Positive (n = 19)	17.2	ns
HCV RNA	Negative (n = 18)	17.26	-
Transfusion Reaction	Present (n = 5)	81.0	*p* < 0.05
Transfusion Reaction	Absent (n = 41)	21.0	-
Splenectomy + HCV-negative	Yes (n = 4)	Highest observed values	Descriptive only

## 4. Discussion

The findings of this study shed new light on the complex interplay between chronic HCV infection, host immunogenetic background, and atopic manifestations in pediatric patients with beta-thalassemia. Our data indicate that HCV-positive individuals demonstrate significantly lower levels of total IgE and reduced allergic symptomatology when compared to their HCV-negative counterparts. These observations are particularly noteworthy given the well-documented immunological disturbances that characterize polytransfused thalassemic patients, such as chronic antigenic stimulation, altered cytokine profiles, and, in many cases, splenectomy-induced changes in immune surveillance [17,34]. One of the most compelling aspects of our results lies in the apparent association between specific genetic polymorphisms and IgE-mediated immune responses. The overrepresentation of the IL10 −1082 AA and TLR7 rs179008 TT genotypes among HCV-positive patients with low IgE levels suggests a genetically mediated predisposition toward an immunoregulatory phenotype. In contrast, IL4 −589 (C>T) and IFNG +874 (T>A) polymorphisms showed no association with atopy in our cohort. Although these variants have been linked to increased IgE levels and atopy in general populations, their effects may have been attenuated here by chronic infection and transfusion-related immune modulation. Previous studies in general populations have reported that the IL4 −589C>T variant is associated with enhanced IL-4 transcription and increased atopic susceptibility, while IFNG +874T>A influences interferon-gamma production and Th1/Th2 balance. In our cohort, however, the potential impact of these variants may have been masked by strong environmental and disease-related modulators, including chronic HCV infection and splenectomy [35]. IL-10 is a potent anti-inflammatory cytokine capable of downregulating both Th1 and Th2 responses, and it plays a critical role in the establishment of peripheral tolerance. Several studies have demonstrated that IL10 promoter polymorphisms influence cytokine secretion levels, with the −1082 AA genotype generally associated with lower IL-10 production. Paradoxically, our results show that patients carrying this genotype may still exhibit features of immune tolerance, such as low IgE levels and reduced skin test reactivity. This discrepancy may be explained by the context-dependent regulation of IL10 expression, particularly under chronic viral stimulation. Under acute immune activation, IL10 promoter activity is transient and largely governed by feedback mechanisms limiting inflammation. In chronic viral infection, however, prolonged antigenic stimulation and continuous engagement of Toll-like receptors can reshape IL10 transcriptional control. Sustained activation of STAT3 and NF-κB, together with epigenetic modification of the IL10 promoter, has been shown to maintain IL-10 expression despite the presence of genotypes typically associated with low-production phenotypes. This adaptive regulation may explain why the IL10 −1082 AA genotype, although linked to reduced IL-10 output in other settings, corresponds here to a tolerogenic and low-IgE profile [36,37]. The regulatory behavior of IL-10 differs markedly between acute and chronic immune activation. During acute infections, IL-10 acts mainly as a transient anti-inflammatory cytokine that dampens excessive Th1-driven inflammation once pathogen clearance begins. In contrast, chronic antigenic stimulation, such as that induced by persistent HCV infection, leads to sustained IL-10 production through continuous engagement of pattern-recognition receptors and activation of regulatory T-cell pathways. This prolonged IL-10 signaling promotes a tolerogenic environment characterized by Th2 suppression and reduced IgE synthesis.

TLR7, an endosomal pattern recognition receptor, detects single-stranded viral RNA and is highly expressed in plasmacytoid dendritic cells and B cells. Engagement of TLR7 during chronic HCV infection could potentiate type I interferon responses and modulate downstream IL-10 secretion a pathway also implicated in systemic immune modulation [27,38,39]. The rs179008 TT genotype has been linked to altered translation efficiency and receptor function, especially in a sex-specific manner. Though we did not stratify our analysis by sex, future studies should consider this variable given the X-linked nature of TLR7. The apparent protective association between the TT genotype and atopy in our cohort may reflect enhanced viral recognition leading to IL-10 induction and suppression of allergenic Th2 pathways. The hypothesis that chronic HCV infection may induce a tolerogenic immune milieu is supported by prior literature; persistent viral presence often triggers regulatory circuits involving IL-10-producing T cells and a decrease in effector T cell activity a trend echoed in autoimmune-inflammatory pediatric syndromes [29,40,41]. In this context, chronic antigen exposure via HCV may function similarly to allergen-specific immunotherapy, promoting immune deviation or suppression rather than exacerbation. Interestingly, our data also reveal that HCV-negative patients, especially those who are splenectomized, display elevated IgE levels and more frequent allergic symptoms. This further supports the notion that, in the absence of viral-induced immune regulation, other factors such as transfusion history, absence of splenic immune modulation, or underlying genetic predispositions may tip the balance toward atopy.

The influence of splenectomy on immune modulation in thalassemia is multifaceted. The spleen plays a vital role in filtering blood-borne pathogens, maintaining B cell homeostasis, and regulating immune tolerance. Splenectomy has been associated with both heightened susceptibility to infection and dysregulated immune responses, including autoimmune and allergic manifestations

In our cohort, splenectomized, HCV-negative individuals exhibited the highest IgE levels and most prominent allergic symptoms. This additive effect suggests that the immunologic consequences of splenectomy may synergize with the absence of chronic viral modulation to promote Th2-driven atopic responses. While our findings are insufficient to draw causal inferences, they highlight the need to account for splenectomy status in studies exploring immune deviation in thalassemia.

It is also important to interpret our data within the limitations of the study design. The small sample size, particularly within the HCV-negative subgroup, precludes definitive statistical conclusions and restricts our ability to conduct robust multivariate analyses. The absence of cytokine profiling, allergen-specific IgE measurements, and flow cytometry-based immune phenotyping limits the mechanistic insights that can be drawn from the observed genotype–phenotype associations.

Furthermore, environmental and treatment-related confounders such as iron chelation therapy, viral coinfections, and nutritional status were not systematically evaluated. These factors can influence both immune function and IgE production and warrant inclusion in future research, as also highlighted in complex diseases [11,27,42,43,44,45,46,47]. Despite these limitations, our findings contribute to a growing body of evidence suggesting that chronic infections can modulate allergic risk. Previous studies in helminth-endemic regions have shown inverse associations between chronic parasitic infection and atopy, attributed to IL-10-mediated immune regulation. Similarly, persistent viral infections like EBV and CMV have been implicated in immune deviation away from allergenic responses. Our study adds to this paradigm by proposing that HCV, in the context of specific host genotypes, may exert a similar modulatory effect in a non-parasitic setting [45,46]. The potential clinical implications of these findings are manifold. If chronic HCV infection indeed promotes a tolerogenic immune state via IL-10 and TLR7 pathways, therapeutic strategies aimed at enhancing such regulatory circuits could be explored in allergic disease. Conversely, the clearance of chronic infections such as HCV with direct-acting antivirals (DAAs) might theoretically unmask underlying atopic tendencies in genetically predisposed individuals, although this remains speculative [39,41,47,48,49,50,51]. Monitoring immune markers before and after DAA therapy in thalassemic patients could offer valuable insights into such dynamics. The cross-sectional design and limited sample size precluded the use of logistic regression or other multivariate models. Consequently, the observed associations between HCV infection, genetic polymorphisms, and atopy should be interpreted as exploratory and not as evidence of independent predictive effects.

## 5. Conclusions

Our findings suggest that in pediatric patients with beta-thalassemia, chronic HCV infection is associated with reduced allergic sensitization and lower serum IgE levels. This effect may be further influenced by genetic polymorphisms in IL10 and TLR7, supporting a model of gene–environment interaction in immune modulation. While preliminary, these data highlight the need for further research into immunogenetic determinants of atopy in chronically exposed pediatric populations. Identification of such factors could aid in stratifying allergy risk and optimizing management strategies in thalassemia and related disorders.

## Figures and Tables

**Table 1:** A summary of the demographic, clinical, and genetic characteristics of participants by HCV status. Comparisons performed using Student’s *t*-test (HCV+ vs. HCV−) and one-way ANOVA (controls vs. BT groups). *p* < 0.05 considered statistically significant.

Group	N	Mean Age (Years)	M:F Ratio	Mean IgE (IU/mL)	Comparison (HCV+ vs. HCV−)	Allergy Symptoms (%)
HCV-positive BT	37	10.1 ± 3.2	21:16	18.73 ± 4.2	*p* < 0.001	21.6%
HCV-negative BT	9	9.8 ± 3.0	5:4	118.76 ± 7.9		55.5%
Healthy controls	50	10.5 ± 2.9	24:21	16.84 ± 5.4	*p* < 0.05 (vs. both BT groups)	22.2%

**Table 2:** Genotype frequencies and their association with atopy across the groups. Given the limited sample size, inferential statistical testing was not performed. Values indicate descriptive genotype trends.

SNP	Genotype	HCV-Positive (n = 37)	HCV-Negative (n = 9)	Atopy Prevalence (%)	Comparison (HCV+ vs. HCV−)
IL10 −1082	AA	23 (62.2%)	3 (33.3%)	13.0% (HCV+) vs. 66.7% (HCV−)	AA higher in HCV+, lower atopy
IL10 −1082	AG/GG	14 (37.8%)	6 (66.7%)	21.4% (HCV+) vs. 33.3% (HCV−)	AG/GG more frequent in HCV−
TLR7 rs179008	TT	19 (51.4%)	2 (22.2%)	10.5% (HCV+) vs. 50.0% (HCV−)	TT enriched in HCV+, lower atopy
TLR7 rs179008	CT/CC	18 (48.6%)	7 (77.8%)	22.2% (HCV+) vs. 28.6% (HCV−)	CT/CC more frequent in HCV−

## Data Availability

The data that support the findings of this study are available from the corresponding author upon reasonable request. Individual participant data are not publicly available to protect participant privacy.

## Abbreviations

The following abbreviations are used in this manuscript

HCV	Hepatitis C Virus
IL10	Interleukin 10
TLR7	Toll-Like Receptor 7
IgE	Immunoglobulin E
Treg	Regulatory T Cell
MIS-C	Multisystem Inflammatory Syndrome in Children
ATP6V1B2	ATPase H+ Transporting V1 Subunit B2
CF	Cystic Fibrosis
ROS	Reactive Oxygen Species
Th	T-helper
